# Longevity Greatest among the Poor

**Published:** 1844-07

**Authors:** 


*Longevity greatest among the poor.*—We will lastly allude to the extraordinary fact, as noticed by all writers on centenarians, that they are generally, if not universally, found in that class of society which gives the lowest mean duration of life. M. Corbeaux, who made researches upon the English and Scotch centenarians, says—"The recorded instances of unusual longevity are, almost without exception, confined to old soldiers who have escaped the worst chances of war, and to peasants whose circumstances placed them but a single degree above want of the common necessaries." In all Belgium, on the 1st of January, 1831, there were but sixteen centenarians, all of whom, according to Quetelet and Smets, belonged to the same class of population, *y vivaient dans des conditions trés médiocres.*

It is thus seen from the preceding researches, as regards centenarians, that they are most apt to be found in the worst periods of man's history, as respects the condition of population, and often too in the most insalubrious localities; that they abound in countries having a low mean duration of life, as in Geneva, in the sixteenth century, and that they decrease and finally disappear in proportion as the mean term of man's existence is extended by the improvement of his physical condition; and lastly, that they are always found in that class of society, which has the lowest mean duration of life. Hence it follows conclusively that a high proportion of centenarians does not depend upon a protracted mean duration of life in a community, nor is a great proportion of them to be regarded as an indication of national health or general longevity.

It is thus satisfactorily shown that of all attempts hitherto made by a certain class of philosophers to establish a distinction of species between the Caucasian and African, the present one, based upon the ratio of centenarians, is truly the most absurd. It also teaches the moral that he, who would strike a blow at one of the great principles of civilization, not to say of the christian religion, should first, at least through self-respect, make himself acquainted with what is known upon the subject.

Dr. Forry, in *N. Y. Jour. Med.*, for May, 1844.

